# Diverse Planning for UAV Control and Remote Sensing

**DOI:** 10.3390/s16122199

**Published:** 2016-12-21

**Authors:** Jan Tožička, Antonín Komenda

**Affiliations:** AI Center, Department of Computer Science, Faculty of Electrical Engineering, Czech Technical University in Prague, 166 27 Praha 6, Czech Republic; antonin.komenda@fel.cvut.cz

**Keywords:** diverse planning, UAV, remote sensing

## Abstract

Unmanned aerial vehicles (UAVs) are suited to various remote sensing missions, such as measuring air quality. The conventional method of UAV control is by human operators. Such an approach is limited by the ability of cooperation among the operators controlling larger fleets of UAVs in a shared area. The remedy for this is to increase autonomy of the UAVs in planning their trajectories by considering other UAVs and their plans. To provide such improvement in autonomy, we need better algorithms for generating alternative trajectory variants that the UAV coordination algorithms can utilize. In this article, we define a novel family of multi-UAV sensing problems, solving task allocation of huge number of tasks (tens of thousands) to a group of configurable UAVs with non-zero weight of equipped sensors (comprising the air quality measurement as well) together with two base-line solvers. To solve the problem efficiently, we use an algorithm for diverse trajectory generation and integrate it with a solver for the multi-UAV coordination problem. Finally, we experimentally evaluate the multi-UAV sensing problem solver. The evaluation is done on synthetic and real-world-inspired benchmarks in a multi-UAV simulator. Results show that diverse planning is a valuable method for remote sensing applications containing multiple UAVs.

## 1. Introduction

Measuring air quality has been historically performed by ground stations. Later on, manned aircraft and satellites were used to collect necessary measurements. Unfortunately, airborne and satellite sensors are very costly which prevents their daily use. Most recently, remotely controlled unmanned aerial vehicles (UAVs) equipped with different sensors are being used to get up-to-date information with higher spatial and temporal resolution at reasonable equipment price. The use of UAVs for air quality monitoring is getting more and more attention from both the research community and industry. The most common usage of UAVs are air pollution and emission monitoring [1], climate change monitoring [2], emergency response [3], disaster monitoring (e.g., forest fires [4,5] or chemical factory explosions [6], etc.), area monitoring [7], or wildlife monitoring and protection [8,9]. In this work, we focus on autonomous UAVs which could collect required data in a coordinated manner without any human aid.

The reason to move from remotely controlled UAVs to autonomous UAVs is the fact that human operators (pilots) seem to be a bottleneck of the system when several UAVs collaborate on a single mission [10]. Each operator or a team of operators is responsible for one UAV and controls its actions. The human operators communicate among themselves and coordinate their actions in order to achieve a common goal. Such an approach has its limits in the human interactions and human control of the UAVs. Therefore, one of the main goals of research tackling UAVs is to improve management of the UAVs such that an operator or a group of operators can control larger groups of UAVs easily. This can be achieved by two means:

- improve human–machine interface (HMI),
- increase UAV autonomy.

In this article we provide a follow-up to our previous work in [10,11,12,13] on advanced Human-Machine Interfaces (HMIs) using planning of alternatives aimed at the first approach and explore solutions tackling the second approach with the help of planning of alternative plans as well. We have already demonstrated how multiagent control algorithms can be used to control multiple UAVs [14]; therefore, the method proposed in this article also continues in this direction and provides methods of improving UAV planning capability by utilization of planning for alternatives.

**Figure 1:**
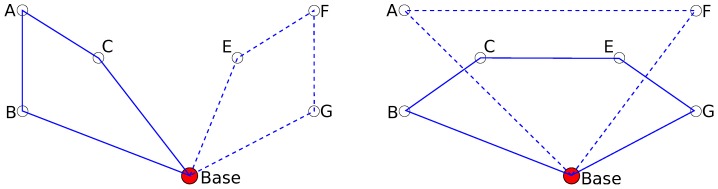
Example of planning alternative trajectories of two unmanned aerial vehicles (UAVs) for a task of covering a set of waypoints (A–G). Two possible solutions are shown for both UAVs (solid and dashed lines).

An illustrative example of planning alternatives for trajectory planning is shown in Figure 1. There are two UAVs and six waypoints that need to be visited by either UAV. Planning of alternatives can propose several possible solutions to the task. Two of them are shown in the figure.

Unlike in the case of making alternative plans for human operators, where the utility function defining the quality of the solution is unknown (or only implicitly known only to the operator) in the case of fully autonomous UAVs, the utility function is known but the optimization problem is too complex to be solved optimally. Our proposed approach here is to use planning of alternatives to provide a diverse set of trajectories out of which final trajectories for all the UAVs are chosen. Since the set of created diverse trajectories is processed automatically, the size of the created set can be much bigger than when we want a human operator to choose. We named out approach *diverse planning*.

Although the solution is not limited to it, our task in this work is to monitor different air pollutants across a city. Even though the dense monitoring is necessary to detect sources of the pollution, it is rather an overkill for uniform continuous monitoring. Different pollutants are required to be monitored at different locations. For example, near a main road junctions, it is necessary to monitor gases produced during combustion: carbon dioxide (CO${}_{2}$), methane (CH${}_{4}$), and nitrous oxide (N${}_{2}$O), while near schools it is necessary to monitor ultrafine particles [15], carbon monoxide (CO), and sulfur dioxide (SO${}_{2}$), which negatively affect human health [16].

A UAV or a team of UAVs can be also used for monitoring of remote and inaccessible areas, as proposed, e.g., in [1,17,18,19]. In our case, we want to monitor a city (particularly, we are using simulation for the city of Prague), which requires low altitude flights (disallowing monitoring by conventional aircraft). There is a full range of UAVs which could be used for this task (Provided that the legal and regulation issues [20,21] are solved). These UAVs differ in their sizes, range of flights, payload and power capacities, speeds, etc. In this study we do not focus on any particular UAV and the proposed method can be easily used for any type of UAV by correctly setting few basic parameters. For our purpose we could use, for example, *Meteorological Mini-UAV* (*M2AV*) developed at the Institute of Aerospace Systems, Technical University of Braunschweig, Germany. The maximum take-off weight is 4.5 kg, including 1.5 kg of payload, with the range of 60 km at a cruising speed 20 ms${}^{-1}$.

The presented task here is a real-world-inspired application called Multi-UAV Sensing Problem (MUSP). The goal is to gather air quality data for a large area using a group of UAVs. Different types of locations are required to be monitored by different sensors. Each UAV can be equipped with multiple sensors, but the weight of each mounted sensor negatively affects the fuel amount which can be carried and thus limits UAV flight range. As the authors in [1] mentions:

[R]ealistically even the lightest onboard sensors would add some weight. The heavier the payload the less fuel can be added, which reduces flight duration.

This overview article implies that our algorithm is the first one considering non-zero sensors weights at such a scale. The solution of the MUSP has to specify which sensors have to be mounted on which UAV and also plan the flight trajectory which has to be shorter with each mounted sensor. The quality of the solution is measured by the total number of performed measurements.

In MUSP, we can use diverse planning to provide a set of diverse trajectories out of which the most suitable trajectories for the UAVs are chosen. This approach allows us to balance the quality of the solution and the required computational time, which is necessary for large-scale applications.

The article is structured as follows. In Section 2, we formally define the problem of multi-UAV coordination for remote sensing with two base-line solutions using the classical and greedy planning techniques. In Section 3, we present the novel algorithm DivPlan based on diverse planning techniques providing efficient solution to the defined family of remote sensing problems. In Section 4, we provide a complexity analysis for the three algorithms. Finally, we experimentally compare the proposed algorithm with the two base-line approaches in Section 5 and also evaluate the algorithm in simulation of a real-world problem.

## 2. Diverse Planning for Multi-UAV Coordination

The diverse planning techniques can be used directly for improved interaction with a human UAV operator, but also for algorithms planning for a team of UAVs aiming at improved autonomous behavior. Before presenting the Multi-UAV coordination planner coined DivPlan utilizing diverse planning, we present two base-line algorithms. Since the output of the algorithms are trajectories in the form of GPS coordinates it is very simple to deploy them on any UAVs supporting flight along GPS trajectories, e.g., using mixed-reality system as have been demonstrated in [14].

Firstly, we will present an pseudo-optimal planner based on translation of the problem to classical optimal planning; Secondly, we will present a greedy approach. On one hand, the pseudo-optimal algorithm provides solutions close to optima; however, at the price of high (generally intractable) computational complexity. On the other hand, the greedy approach is computationally easy (tractable); however, the solutions are often of low quality. The motivation for DivPlan was to design a middle-ground algorithm with complexity low enough for large-scale scenarios; however, with solutions of higher quality than the naive greedy approach.

The Multi-UAV Sensing Problem (MUSP) is defined as a tuple M=〈Y,L,U,T,c,b,p〉, where

- y∈Y is a set of sensor types the UAVs can equip in form of particular sensors,
- $\bar{l}\in L$ is a set of target ground locations for sensing in form $\bar{l}=\langle l_{x},l_{y}\rangle$,
- u∈U is a set of (identificators of) the UAVs carrying out the mission,
- $\langle \bar{l},y\rangle \in T$ is the set of the sensing tasks of the UAVs of sensor type *y* at target location $\bar{l}$ (the optimization criterion is to fulfill maximal number of these tasks),
- *c* is the number of sensor slots (identical for all UAVs),
- *b* is the maximal battery charge (identical for all UAVs), and
- *p* represents the battery penalty for one equipped sensor (identical for all UAVs).

The semantics of fulfilment of a task $\langle \bar{l},y\rangle$ is following. The task has to be fulfilled by a UAV located at $\bar{l}$ with an equipped sensor of type *y*. Although each vehicle can be equipped by a number of sensors (maximally *c*), the more sensors attached, the heavier the vehicle is, therefore the smaller is its flight range. The decrease of the flight range is defined by decrease of the maximal (and initial) battery charge by equipping sensors on the vehicle. The reduced initial charge is b−pe for *e* equipped sensors.

A solution of a MUSP problem is a mapping μ:u↦〈traj,eqSensors〉, where for each UAV u∈U a trajectory traj and a set of equipped sensors of types y∈Y in eqSensors are assigned. A trajectory is an ordered sequence of locations from *L* (or an empty sequence) over which the UAV moves to fulfill the tasks. For the length of the trajectory (the distance between two locations $\left|\right|{\bar{l}}_{1}-{\bar{l}}_{2}\left|\right|$ is computed as Euclidean distance), it must hold that the battery charge b−pe is sufficient (we assume WLOG that the units of battery charge are the same units as the distance). For the number of equipped sensors, it must hold |eqSensors|≤c. An optimal solution *μ* of a MUSP problem M is such that there exists no other solution $\mu^{\prime }$ to M fulfilling more tasks $\langle \bar{l},y\rangle \in T$ than *μ*. The closer is a solution to the optimum (to the maximal number of solved tasks), the higher is the quality of the solution.

After we sketch why MUSPs are hard to solve in Section 2.1, we show how to solve the discretized variant of the problem optimally in Section 2.2. The second algorithm, presented in Section 2.3, is the greedy solution.

### 2.1. Why Is This Task Difficult?

A MUSP combines several well-known NP-complete problems; however, to our best knowledge this particular combination has not been proposed and formally defined yet.

For one UAV, one sensor type and the case when it is possible to fulfill all tasks, the problem reduces to a Travelling Salesman Problem (TSP). The selection of sensors under the limit of the battery thus flying distance is a Knapsack Problem (KP). The combination of TSP and KP is know as Orienteering Problems (OP) [22]; however, defined over different prices of the goals, whereas we limit the total flight range in our problem. The MUSPs additionally adds the combinatorial problem of optimization for multiple UAVs.

### 2.2. The Pseudo-Optimal Algorithm

The (close to) optimal solution to a MUSP will be obtained by translating the problem to a classical planning problem and using top-performing optimal planner SymBA* [23] to search for a solution. Detailed description of translation MUPS into a planning problem can be found in the Appendix A. The solution is than translated to *μ* by means of prescription which UAV should use which sensors and how to move among the targets and which to sense. As classical planning does not directly allow modeling of continuous fluents, the proposed translation uses discretization of distances between the locations of sensor tasks and related values as battery charge. Although the discretization causes the optimal solution to the translated problem does not necessarily corresponds to optimal solution to the original MUSP the error is bounded by |T|d, where |T| is the number of sensor tasks and *d* is the distance for one discrete flight “step”, i.e., the discretization factor. Therefore, we denote the algorithm as pseudo-optimal.

### 2.3. The Greedy Algorithm

The *greedy* algorithm sequentially generates and assigns trajectories and equipped sensors to each UAV. The algorithm is listed as Algorithm 1. Each UAV is assigned a trajectory and sensors by a method GreedyOP listed as Algorithm 2. Sensor tasks covered by created trajectory are removed from the problem before the creation of another trajectory for the next UAV.
**Algorithm 1:**
GreedySolver
(M) – a greedy algorithm solving MUSPs.
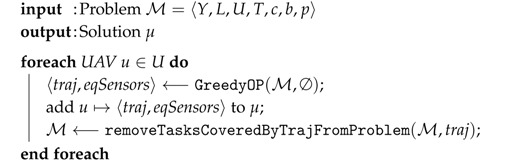


Input of the method GreedyOP (Algorithm 2) is a list of sensor tasks and a list of equipped sensors (this parameter contains no sensor when called from Greedy algorithm, but it will be needed later in the DivPlan algorithm). In fact, GreedyOP solves an Orienteering Problem (OP) together with selection of a suitable subset of sensors. Since the Orienteering Problem is currently an open problem, the proposed solution is another greedy approach. As soon as a practical solution to this problem exists, we shall replace GreedyOP method by a stand-alone solver.
**Algorithm 2:** Greedy Orienteering Problem solver (with greedy sensors selection) of one UAV.
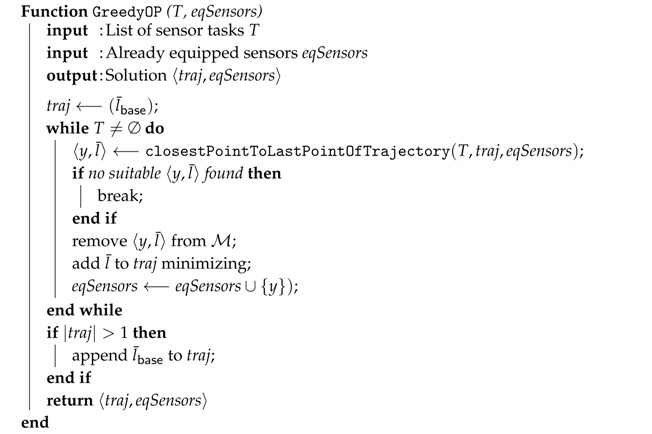


GreedyOP firstly creates an empty trajectory containing only the location of the base ${\bar{l}}_{\mathrm{base}}$ and then it sequentially adds new points to the trajectory as long as the UAV has enough battery charge to return to the base. New points are selected by the method closestPointToLastPointOfTrajectory(T,traj,eqSensors), which finds the closest task of *T* to the last point of the traj. All tasks requiring new sensor (not yet equipped) are penalized by *p* of M and thus tasks with available sensors are preferred. There is also a limit on maximal number of sensors equipped by a single UAV. The semantics of *adding $\bar{l}$ to traj minimizing* is that the new waypoint is added to the trajectory such that extension of the trajectory length is minimized.

The greedy method represents a fast algorithm with prospectively lower solution quality that is supposed to solve large MUSP instances, which cannot be solved by the pseudo-optimal algorithm.

## 3. Diverse Planning Based Algorithm

So far, we presented two algorithms. On one hand, a pseudo-optimal planner which can solve MUSPs nearly optimally but it can solve only very small instances. On the other hand, it is the greedy algorithm which is able to solve large problem instances but since it is a greedy method, it can produce substantially sub-optimal solutions. The main goal of the proposed planner based on diverse planning DivPlan, is to create better solutions than greedy, but in a reasonable time.

DivPlan in Algorithm 3 works in two phases. Firstly, it uses a diverse planning technique to create diverse set of possible trajectories for the UAVs together with a set of sensors it should be equipped with. Then it assigns a subset of these trajectories to individual UAVs by translation to a Constraint Optimization Problem (COP) [24].
**Algorithm 3:** DivPlan—A MUSP solver based on diverse planning and constraint optimization. **input** : Problem M=〈Y,L,U,T,c,b,p〉 **output**: Solution *μ* $\mu^{\mathrm{greedy}}⟵\mathrm{GreedySolver}\left(M\right)$; $\bar{\mu }⟵\mathrm{CreateDiverseTrajecotries}\left(M\right)\cup {⋃}_{u\in U}\mu^{\mathrm{greedy}}\left(u\right)$; $\mu ⟵\mathrm{COPSolver}(X\leftarrow U,\forall X_{i}\in X:D_{i}\leftarrow \bar{\mu },C,f_{\mathrm{opt}},\mu^{\mathrm{greedy}})$; //
(UAVs *U* as COP variables *X*, //
$\bar{\mu }$ as the COP domains $D_{i}$ for all variables, //
constraints *C* forbidding selection of the same trajectory by two UAVs, //
$f_{\mathrm{opt}}$ maximizing the number of sensor tasks covered by the solution *μ*, //
$\mu^{\mathrm{greedy}}$ as the initial solution, and //
returns *μ* assigning a value from $D_{i}$ for each $X_{i}$, i.e., trajectories to UAVs( **return**
*μ*;

The strategy for creating the diverse trajectories is inspired by the experimentally most successful method for diverse planning [10], i.e., looking for good solutions of modified problems.

Method CreateDiverseTrajecotries (Algorithm 4) creates internally many smaller instances of the MUSP problem containing different subsets of sensor tasks and collects their solutions. The subsets of the sensor tasks are created by clustering method *k*-means [25], which groups tasks that are nearby and thus could be possibly covered by a single UAV. Number of clusters varies from 1 to number of sensor slots *c*, generating trajectories for various numbers and combinations of sensors. Then the algorithm repeatedly calls GreedyOP method until all tasks of the cluster are covered. This process is repeated for all subsets of sensors that can be mounted on an UAV and all the created trajectories are stored together with these required sensors.

Once a large portfolio of diverse trajectories is created, DivPlan runs a COP solver. We use OptaPlanner (http://www.optaplanner.org/) a popular Constraint Satisfaction and Optimization Solver. OptaPlanner assigns a trajectory with a set of sensors 〈traj,eqSensors〉 to each UAV optimizing the selection by $f_{\mathrm{opt}}$. Such assignment solves the original MUSP problem. There is only one rule specifying the quality of the solution, i.e., how many sensor tasks are covered by this solution. The rule in form of optimization criterion follows

$${\arg\max}_{\mu }|T^{\prime }\left|,T^{\prime }\subseteq Ts.t.\forall \langle y,\bar{l}\rangle \exists u:\langle y,\bar{l}\rangle \in T^{\prime },\bar{l}\in traj,y\in eqSensors\right|\langle \mathrm{traj},\mathrm{eqSensors}\rangle =\mu \left(u\right),$$

meaning the maximized number of sensor tasks $T^{\prime }\subseteq T$ has to be covered by the solution *μ*. The rule is also listed in Algorithm algDrool in the Drools syntax (https://docs.jboss.org/drools/release/5.2.0.Final/drools-expert-docs/html/ch05.html) used by OptaPlanner. Note that the trajectory length is not being optimized by OptaPlanner, only the number of covered tasks. For practical purposes, one of the very convenient features of OptaPlanner is that it is an any-time algorithm and thus it produces better solutions as it is granted more computation time.
**Algorithm 4:** Creates a set of diverse trajectories.
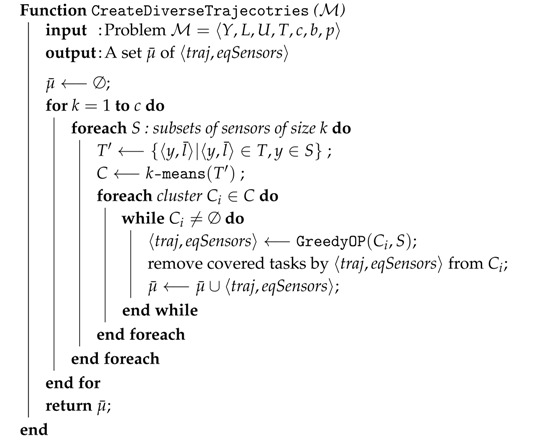

**Algorithm 5:**
OptaPlanner rule (in the Drools syntax). rule ‘‘CoveredTasks’’   when     $task : SensorTask()     not UavPlan(hasSensor($task.sensor), trajectory.isCovered($task.point))   then     scoreHolder.addMediumConstraintMatch(kcontext, −1); end

## 4. Complexity Analysis

A MUSP is combination of several NP-hard problems and thus it is NP-hard. In practice, that means that every algorithm solving this problem optimally needs time growing exponentially with the problem size, unless P = NP. The size of MUSP is dependent on several parameters: number of UAVs |U|, number of sensing tasks |T|, number of different sensors |Y|, and number of sensor slots *c* on a UAV. The number of locations is never more than |L|+1, with +1 for the base location. The maximal battery charge *b* and penalty *p* only limit the number and size of the solutions. Let us take a closer look at how these parameters influence the computational complexity of presented algorithms.

### 4.1. Pseudo-Optimal Algorithm

The pseudo-optimal algorithm works in three steps, translating a MUSP to a classical planning problem, solving the translated problem by a classical planner and back translating the classical plan to the solution of the MUSP instance.

The number of planning objects used in the translation step is $n=\left|L\right|+1+\left|U\right|+c|U|+\left|Y\right|+\frac{b}{d}$, where *d* is the discretization factor. The process of grounding generates all possible parameterizations of the predicates based on the objects of the particular types. Classical planning assumes finite number of objects, therefore the grounding will be finite as well. As all the predicates are binary the asymptotic complexity of grounding of predicates will be $O(n^{2})$. Similarly, grounding of operators generates all possible parameterized actions. As the maximal number of predicate parameters is six for the operator equip, the asymptotic complexity of grounding of operators is $O(n^{6})$. Encoding of the initial state and goal conditions is $O(n^{2})$, because only a subset of facts is used. This gives us polynomial asymptotic complexity for the translation process $O(n^{6})$.

Computational complexity of classical planning (therefore also of the used SymBA* planner) grows in the worst case exponentially with the size of the input problem |Π|. The problem created during the translation is bounded by $O(n^{6})$. Therefore the overall complexity of the solution is exponentially dependent on the input size as follows:

$$O(\exp\left(n^{6}\right)),$$

where the back translation process only linearly traverses the resulting plan and builds the MUSP solution *μ*, therefore there are no additional factors.

It is obvious that this approach is viable for smallest instances only. As we will show in the experiments only up to a dozen of monitoring tasks.

### 4.2. Greedy Algorithm

The greedy algorithm sequentially creates trajectories for each UAV. To create one trajectory, it repeatedly selects sensor tasks and adds the closest one to the existing trajectory. Thus, the whole computation runs in time:

$$O(|U|\cdot {\left|Y\right|}^{2})=O\left(n^{3}\right),$$

which is polynomial in the size of the MUSP instance.

### 4.3. DivPlan Algorithm

DivPlan firstly creates a set $\bar{\mu }$ of diverse trajectories. Number of these trajectories can be estimated directly form Algorithm 4.

|μ¯|≤∑k=1c|eqSensors|k·∑i=1ktraj(Ci),

where $\mathrm{traj}(C_{i})$ is number of trajectories created from cluster $C_{i}$. In the worst case, the $\sum_{i=1}^{k}\mathrm{traj}\left(C_{i}\right)$ can approach the total number of sensor tasks (each trajectory covers just one location with one sensor task), but in practice this number is typically much smaller especially for large number sensor tasks. We can also limit this number by a constant *t*, then:

(1) |μ¯|≤∑k=1c|eqSensors|k·k·t≤t·∑k=1|eqSensors||eqSensors|k·k≤

(2) $$\begin{array}{ccc} & \leq & t\cdot \left|eqSensors\right|\cdot 2^{\left|eqSensors\right|}. \end{array}$$

Hence we bound the total number of diverse trajectories by *t*, the number of diverse trajectories created for one cluster of sensor tasks, and the total number of sensors, is in practice limited.

OptaPlanner is an anytime algorithm and thus it is difficult to evaluate its time complexity, moreover there is too many variables to theoretically estimate its performance profile (for experimental evaluations refer to the following experimental section, particularly Figure 7). Nevertheless, we can evaluate the total size of the search space as

$$|\bar{\mu }{|}^{\left|U\right|}\leq t^{\left|U\right|}\cdot {\left|eqSensors\right|}^{\left|U\right|}\cdot 2^{\left|eqSensors|\cdot |U\right|},$$

which gives us a following asymptotic bound on time complexity of DivPlan:

$$O\left(t^{\left|U\right|}\cdot 2^{c|U|}\right)=O\left(t^{n}\cdot \exp\left(n\right)\right).$$

The time complexity of DivPlan is thus exponentially dependent only on the number of UAVs and their sensor slots. Unlike the number of sensing tasks, these numbers are very limited in practice. If we consider them to be fixed parameters, we would get a polynomial complexity of DivPlan algorithm.

## 5. Experiments

The experimental evaluation compares the three proposed MUSP solvers. The MUSP solvers are evaluated on synthetic benchmarks and in simulated large-scale scenarios. All experiments were performed on 8 core Intel Xeon 2.5 GHz computer with 8GB RAM and Java VM heap size limited to 2 GB.

### 5.1. Comparison of the Multi-UAV Sensor Problem Solvers

We have evaluated more than 3000 different instances of MUSP. In these experiments we compare the three proposed algorithms. Firstly, the pseudo-optimal solver (The problem has to be discretized to be computable in reasonable time, which can cause that the solution is not always the optimal one, therefore pseudo-optimal.) (see Section 2.2 for details). The greedy algorithm represents naive fast algorithm (described in Section 2.3). And finally DivPlan shows how diverse planning together with a COP solver can provide better solution than the greedy algorithm within reasonable time (see Section 3 for details). To compare these algorithms, we designed a set of benchmark instances allowing to scale from a few sensor tasks to tens of thousands. We also demonstrate how the proposed DivPlan method works on a real-world-inspired scenario of monitoring the air pollution in the city of Prague.

The scenario for all synthetic benchmark experiments was created by random generation of a road map and random locations on each road. All locations at the same road were required to be monitored by the same sensor. The whole area was a square 1000 m × 1000 m, the range of flight is 5 km with one mounted sensor and it decreases by 1 km by each additional sensor.

The first set of benchmark tests focuses on the overall solution quality when compared to the optimal solution. These benchmarks contain 3 roads each with 1 to 7 monitoring locations, leading to 3 to 21 sensor tasks. There are 3 types of sensors and each of 2 operating UAVs can hold up maximally 2 sensors.

**Figure 2:**
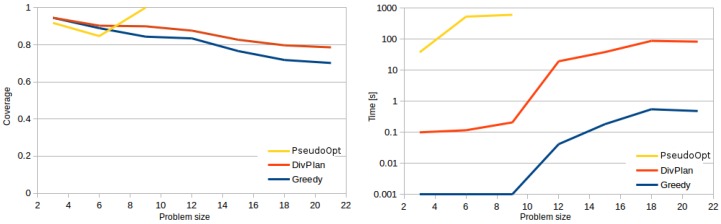
Time and coverage of solved sensor tasks comparison of all methods. Time of DivPlan is time needed to find final solution (DivPlan was always granted 30 min timeout, but typically the final solution has been found within few seconds). Coverage of DivPlan and greedy is always counted for all the problems while for pseudo-optimal it is only over the solved problems. For size 9, the pseudo-optimal planner solved only approximately one fourth of the problem instances. These instances were solved by DivPlan and greedy algorithms with coverage 1 too.

As expected, the results shown in Figure 2 demonstrate that the use of the pseudo-optimal solver (Section 2.2) is impractical for instances containing more than few sensor tasks. In the figure, we can also see that the discretization of the continuous space causes that the result of the optimal solver is in approx. 25% of cases suboptimal. The DivPlan improved the quality of the greedy solution in all but the most trivial cases. The right chart shows that in average DivPlan found solutions in less 100 s while greedy algorithm required less than 1 s.

**Figure 3:**
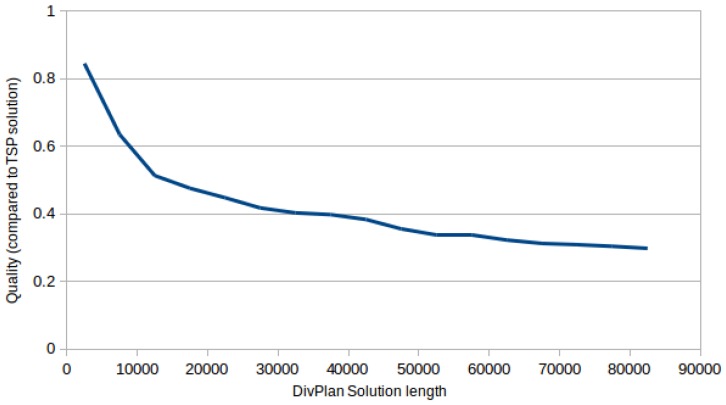
Comparison of DivPlan solution with the optimal solution of “omnipotent UAV” covering the same sensor tasks. We can see that the DivPlan solution was in average at most three times longer even for the longest solutions of scenarios with several dozens of UAVs.

For larger domains with benchmark set containing 1650 problems with up to 2500 sensor tasks, 2 to 10 different sensors, 3 to 5 sensor slots and 10 to 50 UAVs, it is not feasible to find the optimal solution. Nevertheless we would like to have some estimate how good the created solution is. For this purpose we run an optimal TSP solver Concorde (http://www.math.uwaterloo.ca/tsp/concorde.html) on all covered sensor tasks by DivPlan. Its solution then corresponds to the optimal trajectory of one “omnipotent UAV” with all sensors and unlimited flight range, fulfilling the MUSP problem with the same coverage as the solution provided by DivPlan. Figure 3 shows the relative quality of the DivPlan solution (0.5 means that Concorde found trajectory of half length). We can see that even one unlimited UAV would still have to travel at least 30% of the solution length even for the cases containing 40 to 50 UAVs. The average was computed only for problems where Concorde gave a solution within the limit of 10 min.

**Figure 4:**
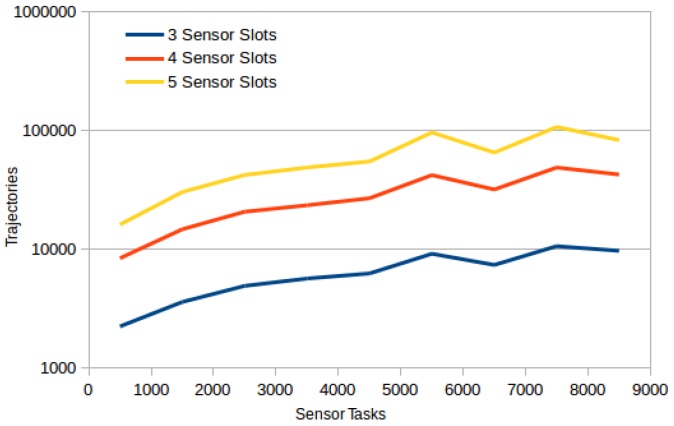
Number of created trajectories for different numbers of sensor tasks for UAVs with 3, 4, and 5 sensor slots. The number of trajectories is in log scale.

The last set of benchmark experiments focuses on the comparison of the greedy algorithm and DivPlan on large problems. The benchmark set contained 1350 problems with up to 9000 sensor tasks, 5 to 20 different sensors, 3 to 5 sensor slots and 10 to 50 UAVs. The Figure 4 shows how many diverse trajectories have been created for different numbers of sensor tasks for UAVs with 3, 4, and 5 sensor slots. We can see that the number of created trajectories grows linearly with the number of sensor tasks beginning with approximately 3000 sensor tasks.

**Figure 5:**
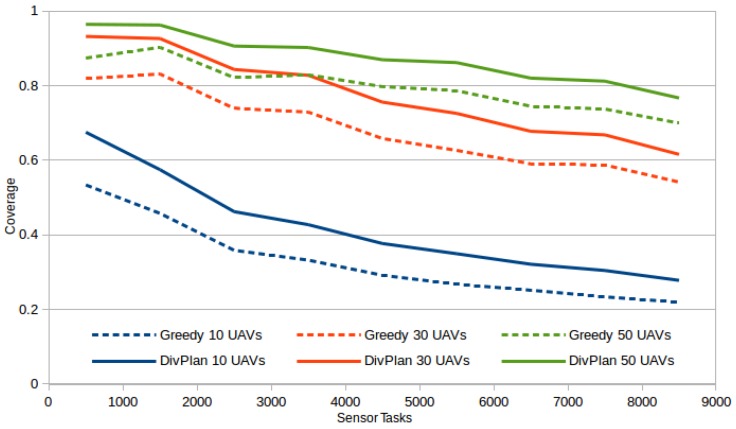
Task coverages for 10, 30 and 50 UAVs on different total number of tasks.

The last chart of this section, Figure 5, shows task coverages for different number of UAVs and different number of sensor tasks. Each line of the graph shows averages over 1060 cases of different settings (number of sensor types, number of sensor slots on UAV, number of roads, etc.). The average improvement is 33% (11 percent points) for 10-UAVs case, 17% (10 percent points) for 30-UAVs case and 12% (8 percent points) for 50-UAVs case. Time limit for DivPlan has been set to 10 min and we can see that it shows a stable improvement over the greedy method for both the different numbers of sensor tasks and the different numbers of UAVs.

### 5.2. Real-World-Inspired Scenario

The motivation for the MUSP problem is monitoring of air pollution in the area 18 km × 16 km of city of Prague. There are 3506 selected locations of 6 different monitoring types, each type is requested to be monitored by 3 different sensors, which yields 10,518 sensor tasks in total. There are 20 UAVs available, each with 5 sensor slots. Since each sensor has non-zero weight, every mounted sensor decreases the UAV range of flight. Table 1 lists the used numbers of equipped sensors and related ranges of flight.

**Figure 6:**
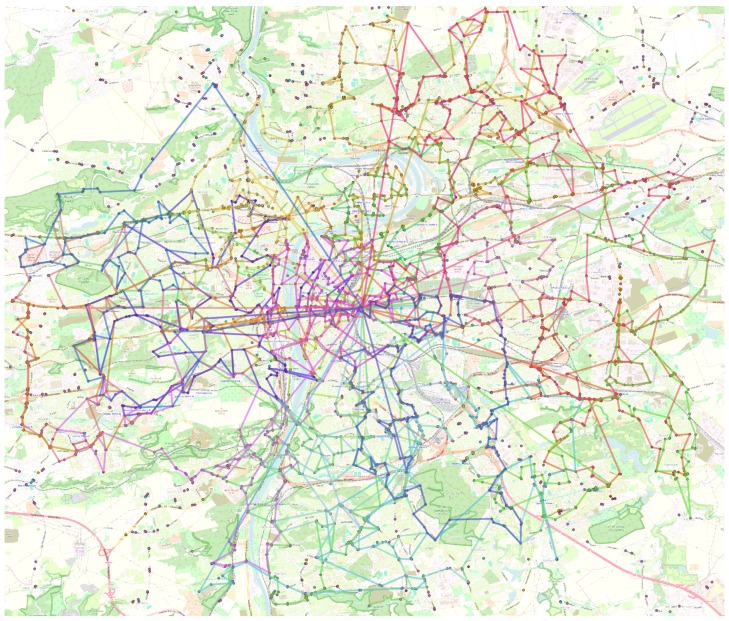
Planned trajectories for 20 UAVs tasked to monitor 3506 locations in Prague. Each location is required to be monitored by 3 different sensors giving 10,518 sensor tasks in total. DivPlan reached coverage of 64%.

**Figure 7:**
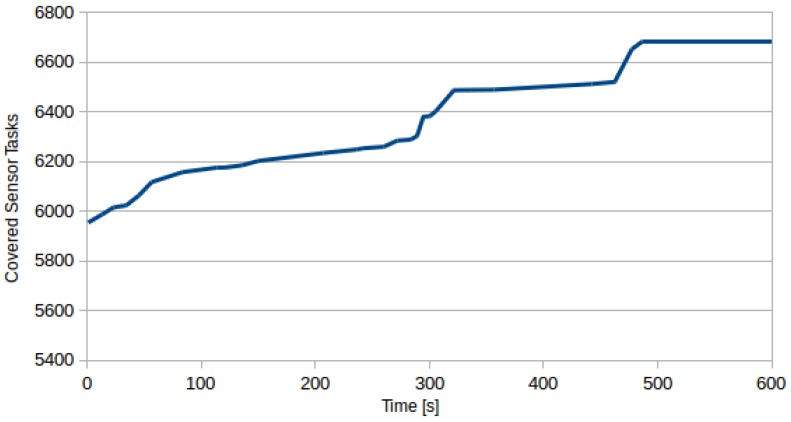
How the coverage improves over time. Base (time 0) is greedy solution: coverage 57%. The time limit has been set to 10 min, but the best solution with coverage of 64% was found after 8.1 min.

Map of monitoring locations together with the UAV plans created by DivPlan are depicted in Figure 6. The greedy solution for this problem provided a solution with sensor task coverage of 57% in 11.5 s. DivPlan improved this solution to the coverage of 64% within 8.1 min. The improvement of the coverage over time is shown in Figure 7.

**Figure 8:**
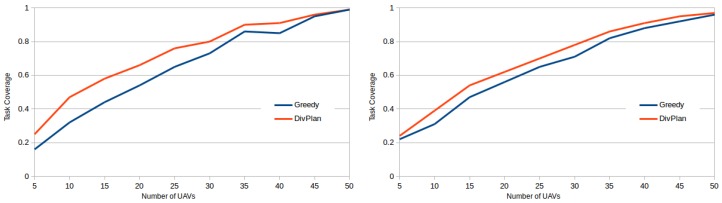
Task coverages for different numbers of UAVs with 3 (left) and 5 (right) sensor slots.

The last graph (Figure 8) compares the task coverage for different numbers of UAVs. We can see that DivPlan improvement over the greedy method was more significant for the case of UAVs with 3 sensor slots.

## 6. Conclusions and Future Work

To solve the problem of autonomous remote sensing with non-zero weight of sensors, we have firstly formally defined the problem and for comparison we have designed two base-line algorithms commonly used for solution of combinatorial optimization problems in the literature. The algorithms were based on two distinct paradigms: (a) translation to classical planning with appropriate discretization; and (b) a greedy approach. The base-line algorithms framed the problem from the perspective of the solution quality and efficiency metrics, respectively.

The main contribution of our work was a novel algorithm aiming at the remote sensing problem by a fleet of coordinated UAVs with practicality in mind. The DivPlan algorithm targets a middle-ground between the optimal but inefficient and low-quality but highly efficient greedy algorithms. To provide such an algorithm, we have integrated an appropriate diverse planning technique to generate the alternative trajectories and Constraint Optimization composing the final solution out of these diverse partial solutions. The solvers were both theoretically and experimentally compared. The results show that an approach based on diverse planning is a good balance between quality of the solution and planning time. Moreover, the greedy and diversity planning approaches were able to solve large problem instances, which demonstrates their good scalability.

Based on the experimental results in the simulated real-world-inspired environment and a conservative usage of waypoints as a robotic primitive, we conjecture DivPlan is a good choice for practical deployment to a Multi-UAV system, which we leave to explore in a future work.

## Figures and Tables

**Table 1:** Decrease of the UAV range of flight based on the number of equipped sensors used in the Real-World-Inspired Scenario.

Number of Equipped Sensors	Range of Flight
1	83 km
2	67 km
3	50 km
4	33 km
5	17 km

**Figure A1:**
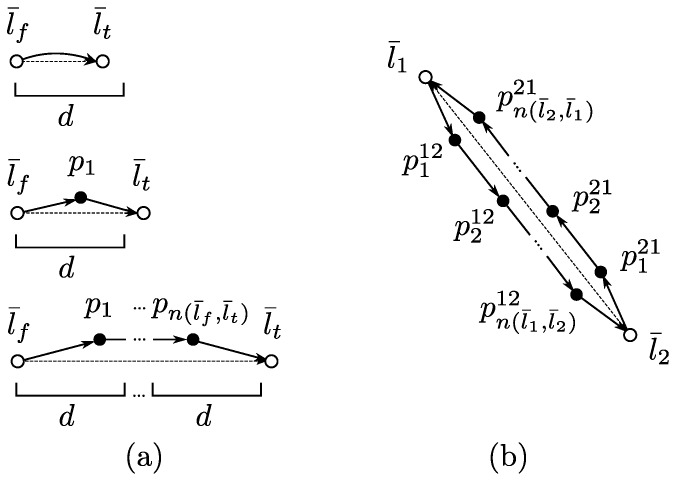
Interpolation of moves between two locations longer than *d* by intermediate positions $p_{i}$. The figure (**a**) shows additional positions $p_{1},\ldots ,p_{n({\bar{l}}_{f},{\bar{l}}_{t})}$ between two locations ${\bar{l}}_{f},{\bar{l}}_{t}$ based on the distance between them and the distance granularity. The solid arrows denote possible move actions. Note that the locations ${\bar{l}}_{f},{\bar{l}}_{t}$ are described by x,y coordinates in the MUSP M, therefore the distance between them (denoted by dashed lines) is defined. On the other hand the positions (in the translated problem), which are either location names or interpolated positions are defined only by means of the objects of type Pos; The figure (**b**) shows interpolation in both directions between two locations ${\bar{l}}_{1},{\bar{l}}_{2}$. The superscripts distinguish direction from ${\bar{l}}_{1}$ to ${\bar{l}}_{2}$ and from ${\bar{l}}_{2}$ to ${\bar{l}}_{1}$.

## Abbreviations

The following abbreviations are used in this manuscript:

MUSP	Multi-UAV Sensing Problem
STRIPS	Stanford Research Institute Problem Solver
TSP	Traveling Salesmen Problem
KP	Knapsack Problem
COP	Constraint Optimization Problem
UAV	Unmanned Aerial Vehicle

## Appendix A. Translation of MUPS to Planning Problem

The discretized distances are interpolated by $\left\lfloor \frac{\left|\right|{\bar{l}}_{1}-{\bar{l}}_{2}\left|\right|}{d}\right\rfloor$ intermediate possible positions of the UAVs. The discretized distances allow us to use also discrete number of battery charge levels $b^{\prime }=\left\lfloor \frac{b}{d}\right\rfloor$. For the discretized battery penalty, we get $p^{\prime }=\left\lfloor \frac{p}{d}\right\rfloor$.

A classical planning problem with costs is defined as a tuple Π=〈F,A,I,G,cost〉 (e.g., in [26]). A set *F* consists in all possible facts which describe states of the modeled world. A state s⊆F contains only such facts that hold in the world in that particular state. A set *A* contains grounded actions a=〈pre(a),add(a),del(a),cost(a)〉, where the sets of facts pre(a)⊆F,add(a)⊆F,del(a)⊆F represent preconditions, add effects and delete effects respectively. The cost function is defined as $\mathrm{cost}:A\to R^{0+}$, where cost(a) represents a non-zero cost of the action *a*. An action can transform a state *s* into a new state $s^{\prime }$ when *a* is executed, provided that all its preconditions are satisfied s.t. pre(a)⊆s and for cost cost(a). The transformation function is defined as $s^{\prime }=\left(s\setminus \mathrm{del}\left(a\right)\right)\cup \mathrm{add}\left(a\right)$. The set I⊆F represents the initial state of the planning problem. The set G⊆F represent goal conditions, such that every state $s_{G}$ for that $G\subseteq s_{G}$ holds is a goal state. Note that the goal condition *G* can represent more conjunctive goal facts which all have to be satisfied. Moreover, the condition allows for satisfaction of the goal facts in various states where additional facts not present in *G* can but need not to hold. A solution to a classical planning problem is a sequence of successively applicable actions beginning in the initial state and ending in one of the goal states, such sequence is called a plan. A sequence of actions $\pi =(a_{1},\ldots ,a_{m})$ is a plan to Π if for all 1<i<m holds $\mathrm{pre}\left(a_{i}\right)\subseteq s_{i}$ and $s_{i+1}=\left(s_{i}\setminus \mathrm{del}\left(a_{i}\right)\right)\cup \mathrm{add}\left(a_{i}\right)$, where $s_{1}=I$ and $G\subseteq s_{m}$.

The sensor tasks in a MUSP are disjunctive. Not all of them can be satisfied if the battery and equipment constraints do not allow it. The objective is defined as an optimization problem, that is we require maximizing of satisfied tasks for the particular M. Moreover, the problem is defined over continuous variables for battery charge, distances and the equipment battery penalty. To solve such problem using a classical planing, we need to appropriately translate it, namely we need to deal with:

1. net-benefit selection of goals (selecting the maximal set of goal facts), and
2. discretization of the continuous variables (locations, moving and battery charge).

Net-benefit planning (sometimes called planning with soft goals) is a well known type of planning problem translation proposed in [27]. The discretization is based on the distance granularity parameter as explained in the previous paragraphs.

The translation will be described in form of parameterized facts, i.e., predicates and parameterized actions, i.e., operators. The initial state and the goal conditions will use parameterized representation as well.

### Appendix A.1. Objects

In classical planning the parameters of predicates and operators are defined in form of typed (mathematical) object. The types used in the translated classical representation of a MUSP are:

- Pos—possible positions of UAVs and sensing targets; each location (in form of its name) $\bar{l}\in L$ is a position, each intermediate interpolation step between two locations is a position as well,
  - Base (Pos subtype)—one of the positions is marked as a base, where the UAVs can equip sensors and where their plans have to begin and end,
- Uav—objects representing the UAVs u∈U
- Slot—each UAV has *c* sensor slots, the objects of type Slot represent such slots,
- Type—types *y* of the sensor targets of the tasks defined as y∈Y and
- Level—number $b^{\prime }$ of possible levels of the UAV battery, the objects of the type Level represent particular charge of a UAV’s battery.

The types define distinct sets of typed objects, therefore we will write x∈SomeType to denote *x* is of a type SomeType.

### Appendix A.2. Predicates

The predicates define parameterized “templates” for the planning facts in *F*. Predicates related to the UAVs are:

- at(Uav,Pos)—in which position a UAV is,
- slotEmpty(Uav,Slot)—whether a slot a of a UAV is not equipped yet,
- senseType(Uav,Type)—whether a UAV is able to sense a sensor type (by equipping appropriate sensor to one of its empty slots) and
- battery(Uav,Level)—current level of the UAV’s battery.

There are three predicates describing object adjacency:

- batteryDec(Level,Level)—relates a battery level to its decremented value by one level (corresponds to depletion of the battery by one move action),
- batteryEquipDec(Level,Level)—relates a battery level to its decremented value by $p^{\prime }$ (corresponds to depletion of the battery by equipping one sensor and models shorter reach by heavier UAV) and
- adj(Pos,Pos) - together with positions describes the movement graph (the move actions are allowed to move only between two adjacent positions).

The tasks $\langle \bar{l},y\rangle \in T$ are described by the last predicate with equal semantics as in the MUSP:

- task(Pos,Type)

By grounding all predicates we get the set *F* of all possible fact (note that not all possible facts form a state, e.g., only one fact grounding battery(Uav,Level) always holds for each UAV).

**Example** **A1.** *For a set of three positions $\left\{p_{1},p_{2},p_{3}\right\}$ and two UAVs $\left\{{\mathrm{uav}}_{1},{\mathrm{uav}}_{2}\right\}$, grounding of the predicate at(Uav,Pos) leads to 6 facts*

$$\begin{array}{c} \mathrm{at}\left({\mathrm{uav}}_{1},p_{1}\right),\mathrm{at}\left({\mathrm{uav}}_{1},p_{2}\right),\mathrm{at}\left({\mathrm{uav}}_{1},p_{3}\right),\\ \mathrm{at}\left({\mathrm{uav}}_{2},p_{1}\right),\mathrm{at}\left({\mathrm{uav}}_{2},p_{2}\right),\mathrm{at}\left({\mathrm{uav}}_{2},p_{3}\right), \end{array}$$

*where a valid part of a state representing positions of the two UAVs is e.g., $\left\{\mathrm{at}\left({\mathrm{uav}}_{1},p_{2}\right),\mathrm{at}\left({\mathrm{uav}}_{2},p_{3}\right)\right\}$.*

An example of a diamond-shaped movement graph with four nodes $\left\{p_{1},p_{2},p_{3},p_{4}\right\}$ would be represented as:

$$\begin{array}{c} \mathrm{adj}\left(p_{1},p_{2}\right),\mathrm{adj}\left(p_{2},p_{3}\right),\mathrm{adj}\left(p_{3},p_{4}\right),\mathrm{adj}\left(p_{4},p_{1}\right),\mathrm{adj}\left(p_{1},p_{3}\right),\\ \mathrm{adj}\left(p_{2},p_{1}\right),\mathrm{adj}\left(p_{3},p_{2}\right),\mathrm{adj}\left(p_{4},p_{3}\right),\mathrm{adj}\left(p_{1},p_{4}\right),\mathrm{adj}\left(p_{3},p_{1}\right). \end{array}$$

#### Operators

The translated actions will be defined in form of parameterized operators. Let us recall the scheme of an action a=〈pre(a),add(a),del(a),cost(a)〉. For definition of the translation operators, we will use parameters of the defined types similarly as for the predicates. The same grounding principle used from predicates to facts will be used for grounding actions from operators.

The first operator represents equipping of a sensor by a UAV:

equip(Uav,Base,Slot,Type,Level,Level).

The grounded actions from the operator not only mark the UAV *u* being able to sense the type *y* by the fact senseType(u,y) (together with removing the fact slotEmpty(u,o), which is required in the preconditions ensuring the sensor slot *o* is not equipped yet). The operator also decrease the current battery level of *u* described by $\mathrm{battery}(u,l_{i})$ to $\mathrm{battery}(u,l_{i+1})$, where the two levels are related by the $p^{\prime }$ decrement in form of the decrement relation facts $\mathrm{batteryEquipDec}(l_{i},l_{i+1})$. The preconditions bind the battery levels and the position of the UAV to the base, as p∈Base and the preconditions contain at(u,p). Complete description of the actions follows:

$$\begin{array}{ccc} \mathrm{pre}(\mathrm{equip}\left(u,p,o,y,l_{i},l_{i+1}\right)) & = & \mathrm{at}\left(u,p\right)\wedge \mathrm{slotEmpty}\left(u,o\right)\wedge \mathrm{battery}\left(u,l_{i}\right)\wedge \\ & & \wedge \mathrm{batteryEquipDec}(l_{i},l_{i+1}),\\ \mathrm{add}(\mathrm{equip}\left(u,p,o,y,l_{i},l_{i+1}\right)) & = & \mathrm{senseType}\left(u,y\right)\wedge \mathrm{battery}\left(u,l_{i+1}\right),\\ \mathrm{del}(\mathrm{equip}\left(u,p,o,y,l_{i},l_{i+1}\right)) & = & \mathrm{slotEmpty}\left(u,o\right)\wedge \mathrm{battery}\left(u,l_{i}\right),\\ \mathrm{cost}(\mathrm{equip}\left(u,p,o,y,l_{i},l_{i+1}\right)) & = & 1. \end{array}$$

Another operator moves a UAV between two positions:

move(Uav,Pos,Pos,Level,Level).

The semantics is straightforward. Before applying a move action, the UAV *u* is in position $p_{i}$ with current battery level $l_{i}$. The UAV can move only to an adjacent position $p_{i+1}$ ensured by $\mathrm{adj}(p_{i},p_{i+1})$ in the preconditions. The battery decrement is modeled similarly as in equip, but it uses $\mathrm{batteryDec}(l_{i},l_{i+1})$ instead of the $\mathrm{batteryEquipDec}(l_{i},l_{i+1})$ decrement relation. The UAV ends in the position $p_{i+1}$ by adding $\mathrm{at}(u,p_{i+1})$ and deleting $\mathrm{at}(u,p_{i})$. The description of the operator follows:

$$\begin{array}{ccc} \mathrm{pre}(\mathrm{move}\left(u,p_{i},p_{i+1},l_{i},l_{i+1}\right)) & = & \mathrm{at}\left(u,p_{i}\right)\wedge \mathrm{battery}\left(u,l_{i}\right)\wedge \\ & & \wedge \mathrm{adj}\left(p_{i},p_{i+1}\right)\wedge \mathrm{batteryDec}\left(l_{i},l_{i+1}\right),\\ \mathrm{add}(\mathrm{move}\left(u,p_{i},p_{i+1},l_{i},l_{i+1}\right)) & = & \mathrm{at}\left(u,p_{i+1}\right)\wedge \mathrm{battery}\left(u,l_{i+1}\right),\\ \mathrm{del}(\mathrm{move}\left(u,p_{i},p_{i+1},l_{i},l_{i+1}\right)) & = & \mathrm{at}\left(u,p_{i}\right)\wedge \mathrm{battery}\left(u,l_{i}\right),\\ \mathrm{cost}(\mathrm{move}\left(u,p_{i},p_{i+1},l_{i},l_{i+1}\right)) & = & 1. \end{array}$$

To fulfill a sensor task $\langle \bar{l},y\rangle \in T$, a UAV has to use the appropriate sensor at the right place. The operator modeling such situation is:

sense(Uav,Pos,Type).

The operator checks whether the UAV is in the right position at(u,p) and equipped with a sensor able to fulfill the task of type *y* by senseType(u,y). If so, the fulfilled task(p,y) is added:

$$\begin{array}{ccc} \mathrm{pre}(\mathrm{sense}(u,p,y)) & = & \mathrm{at}(u,p)\wedge \mathrm{senseType}(u,y),\\ \mathrm{add}(\mathrm{sense}(u,p,y)) & = & \mathrm{task}(p,y),\\ \mathrm{del}(\mathrm{sense}(u,p,y)) & = & \emptyset ,\\ \mathrm{cost}(\mathrm{sense}(u,p,y)) & = & 1. \end{array}$$

The last operator models skipping a translated task $\langle \bar{l},y\rangle \in T$ which cannot be fulfilled by sense:

skip(Pos,Type).

The principle follows the net-benefit translation of soft goals. A soft goal is a goal which does not need to be fulfilled (in the described translation, all goals are soft as some tasks do not need to be fulfilled). The skip actions therefore model not fulfilling a task task(p,y). Since the problem M is defined as optimization over a subset of tasks, the net-benefit planning models the task selection subproblem. The classical planning problem finds a plan with minimal cost of used actions, therefore the skip actions with cost larger than any possible plan can be used by the planner only if the task cannot be fulfilled by sense. The definition of the operator follows:

$$\begin{array}{ccc} \mathrm{pre}(\mathrm{skip}(p,y)) & = & \emptyset ,\\ \mathrm{add}(\mathrm{skip}(p,y)) & = & \mathrm{task}(p,y),\\ \mathrm{del}(\mathrm{skip}(p,y)) & = & \emptyset ,\\ \mathrm{cost}(\mathrm{skip}(p,y)) & > & O(\exp(|\Pi |)), \end{array}$$

where the cost is larger than any possible solution plan of the problem, as the longest possible plan of a classical planning problem is exponential [28] in the size of the input (Practically, we use a large enough constant). By grounding all the operators with all possible parameters defined by their types, we get the set of all actions *A*.

### Appendix A.3. Initial State and Goal Conditions

The complete sets of facts *F* and actions *A* form foundation of the translated MUSP in classical planning. The sets represent a complete transition system for the used objects originating in M and their interaction based on the classical representation modeling the MUSPs.

To finish the translation of M to Π, we need to encode the initial state *I* and goal conditions *G*. First, all UAVs u∈U begin in the base position. Their battery levels are at maximum $l_{b^{\prime }}$. Their sensor slots *o* are initially empty, therefore they cannot sense any target types yet. Formally, in the initial state holds:

$$\begin{array}{ccc} \forall v\in \mathrm{Uav},p\in \mathrm{Base} & : & \mathrm{at}(u,p),\\ \forall v\in \mathrm{Uav},l_{b^{\prime }}\in \mathrm{Level} & : & \mathrm{battery}(u,l_{b^{\prime }}),\\ \forall v\in \mathrm{Uav},\forall o\in \mathrm{Slot} & : & \mathrm{slotEmpty}(u,o). \end{array}$$

The battery level decrement relation holds for all levels from the maximal level $l_{b^{\prime }}$ to the minimal level $l_{0}$ and the equip decrement follows the linear decrement by $p^{\prime }$, such that the last decrement ends in non-negative battery level (minimally $l_{0}$):

$$\begin{array}{ccc} \forall k\in N,b^{\prime }\geq k>0 & : & \mathrm{batteryDec}(l_{k},l_{k-1}),\\ \forall k\in N,\left\lfloor \frac{b^{\prime }}{p^{\prime }}\right\rfloor >k\geq 0 & : & \mathrm{batteryEquipDec}(l_{b^{\prime }-p^{\prime }k},l_{b^{\prime }-p^{\prime }\left(k+1\right)}). \end{array}$$

The adjacency relation is a complete graph with all positions of type Pos as vertices and $n\left({\bar{l}}_{f},{\bar{l}}_{t}\right)=\left\lfloor \frac{\left|\right|{\bar{l}}_{f}-{\bar{l}}_{t}\left|\right|}{d}\right\rfloor$ interpolated points along the edges (see Figure A1) using the distance granularity *d*. The formal definition follows:

$$\begin{array}{ccc} \forall {\bar{l}}_{f}\in \mathrm{Pos},\forall {\bar{l}}_{t}\in \mathrm{Pos},n\left({\bar{l}}_{f},{\bar{l}}_{t}\right)=0 & : & \mathrm{adj}({\bar{l}}_{f},{\bar{l}}_{t});\\ \forall {\bar{l}}_{f}\in \mathrm{Pos},\forall {\bar{l}}_{t}\in \mathrm{Pos},n\left({\bar{l}}_{f},{\bar{l}}_{t}\right)=1 & : & \mathrm{adj}\left({\bar{l}}_{f},p_{1}\right),\mathrm{adj}\left(p_{1},{\bar{l}}_{t}\right);\\ \forall {\bar{l}}_{f}\in \mathrm{Pos},\forall {\bar{l}}_{t}\in \mathrm{Pos},n\left({\bar{l}}_{f},{\bar{l}}_{t}\right)>1,\\ \forall k\in N,n\left({\bar{l}}_{f},{\bar{l}}_{t}\right)>k>0,p_{k}\in \mathrm{Pos},p_{k+1}\in \mathrm{Pos} & : & \mathrm{adj}(p_{k},p_{k+1}),\\ & & \mathrm{adj}\left({\bar{l}}_{f},p_{1}\right),\mathrm{adj}\left(p_{n({\bar{l}}_{f},{\bar{l}}_{t})},{\bar{l}}_{t}\right). \end{array}$$

Recall the move action decreases the battery by one level. The interpolation therefore splits longer distances than *d* between locations by moves with an error maximally *d*, which correspond to decrease of one battery level.

The goal conditions *G* contain all required tasks $\langle \bar{l},y\rangle \in T$ in the form of conjunction of facts task(Pos,Type) with the position corresponding to he location $\bar{l}$:

$$\begin{array}{ccc} \forall \langle \bar{l},y\rangle \in T & : & \mathrm{task}(\bar{l},y). \end{array}$$

A optimal solution to Π can be straightforwardly translated to a solution of the original MUSP M by mapping of the equipped sensors and moves into the trajectories.

**Example** **A2.** *Let us have an example translated problem with two UAVs $\left\{{\mathrm{uav}}_{1},{\mathrm{uav}}_{2}\right\}$ each with two sensor slot ${\mathrm{slot}}_{1},{\mathrm{slot}}_{2}$, initially at the* *base* *location, two sensor types {a,b} and three tasks {task(left,a),task(left,b),task(right,a)}. The positions representing the locations are interconnected by the interpolated moves with different battery level costs. Moving from the left position to the base position and vice versa costs 4 battery levels and the two other moves 3 battery levels. Provided that $b^{\prime }$ and $p^{\prime }$ allows for a complete solution by one UAV (e.g., $b^{\prime }=12$ and $p^{\prime }=1$), the resulting plan is in form:*

$$\begin{array}{ccc} & & \mathrm{equip}\left({\mathrm{uav}}_{1},\mathrm{base},{\mathrm{slot}}_{1},a,l_{12},l_{11}\right),\mathrm{equip}\left({\mathrm{uav}}_{1},\mathrm{base},{\mathrm{slot}}_{2},b,l_{11},l_{10}\right),\\ & & \mathrm{move}\left({\mathrm{uav}}_{1},\mathrm{base},p_{1}^{\mathrm{base},\mathrm{right}},l_{10},l_{9}\right),\ldots \mathrm{move}\left({\mathrm{uav}}_{1},p_{2}^{\mathrm{base},\mathrm{right}},\mathrm{right},l_{8},l_{7}\right),\\ & & \mathrm{sense}({\mathrm{uav}}_{1},\mathrm{right},a),\\ & & \mathrm{move}\left({\mathrm{uav}}_{1},\mathrm{right},p_{1}^{\mathrm{right},\mathrm{left}},l_{7},l_{6}\right),\ldots \mathrm{move}\left({\mathrm{uav}}_{1},p_{2}^{\mathrm{right},\mathrm{left}},\mathrm{left},l_{5},l_{4}\right),\\ & & \mathrm{sense}\left({\mathrm{uav}}_{1},\mathrm{left},a\right),\mathrm{sense}\left({\mathrm{uav}}_{1},\mathrm{left},b\right),\\ & & \mathrm{move}\left({\mathrm{uav}}_{1},\mathrm{left},p_{1}^{\mathrm{left},\mathrm{base}},l_{4},l_{3}\right),\ldots \mathrm{move}\left({\mathrm{uav}}_{1},p_{3}^{\mathrm{left},\mathrm{base}},\mathrm{base},l_{1},l_{0}\right). \end{array}$$

*This example concludes the appendix on translation of MUSP solving to classical planning.*
